# Supplementary material for: Features of mobile apps for diabetic kidney disease self-management: A scoping review
Source: PLoS One. 2026 Mar 20;21(3):e0331604. doi: 10.1371/journal.pone.0331604 (PMC13004320; doi:10.1371/journal.pone.0331604)
Supplement: S3 Table — (DOCX) [file pone.0331604.s003.docx]

**Supporting information**

**S3 Table: Total Hits for Searching Strategy**

|  |  |  | **TOTAL HITS FOR DATABASE** | | | | |
| --- | --- | --- | --- | --- | --- | --- | --- |
| **NO.** | **THEME** | **TERMS** | **SCOPUS** | **PUBMED** | **SAGE** | **SCIENCE DIRECT** | **WEB OF SCIENCE** |
| 1 | DM-CKD | (diabet* OR T2DM OR T1DM OR TIIDM OR TIDM) AND (chronic AND (kidney OR renal) AND (disease OR failure OR dysfunction OR impairment OR insufficiency OR transplantation OR damage OR problem)) | Not Applicable | | | | |
| 2 | DKD | (diabet* OR T2DM OR T1DM OR TIIDM OR TIDM) AND (nephropath* or "kidney disease") | Not Applicable | | | | |
| 3 | Self management | self-control OR monitor* OR control* OR “self administer*” OR “self treat*” OR “self manag*” OR “self car*” OR “self adhere*” OR “self efficacy” OR self* OR manag* OR care* | Not Applicable | | | | |
| 4 | Mobile apps | mhealth OR m-health OR "Mobile App*" OR "mobile" AND ("device" OR "health*" OR "phone") OR Mobile* OR smartphone* OR tele* OR “Cell* Phones” OR “digital health” OR “mHealth app*” | Not Applicable | | | | |
|  | Combination  of terms | (((diabet* OR T2DM OR T1DM OR TIIDM OR TIDM) AND (chronic AND (kidney OR renal) AND (disease OR failure OR dysfunction OR impairment OR insufficiency OR transplantation OR damage OR problem))) OR ((diabet* OR T2DM OR T1DM OR TIIDM OR TIDM) AND (nephropath* or "kidney disease"))) AND (self-control OR monitor* OR control* OR “self administer*” OR “self treat*” OR “self manag*” OR “self car*” OR “self adhere*” OR “self efficacy” OR self* OR manag* OR care*) AND (mhealth OR m-health OR "Mobile App*" OR "mobile" AND ("device" OR "health*" OR "phone") OR Mobile* OR smartphone* OR tele* OR “Cell* Phones” OR “digital health” OR “mHealth app*”) | 211 | 564 | 211 |  |  |
|  | MESH term | (“Diabetes” AND “Chronic Kidney Disease”) OR (“Diabetes nephropathies”) OR (“diabetic kidney disease”) AND (“self management” OR “Self care”) AND (“Mobile Applications” OR “m-health”) |  |  |  | 4462 | 2536 |
