# Supplementary material for: Features of mobile apps for diabetic kidney disease self-management: A scoping review
Source: PLoS One. 2026 Mar 20;21(3):e0331604. doi: 10.1371/journal.pone.0331604 (PMC13004320; doi:10.1371/journal.pone.0331604)
Supplement: Attachment — Submitted filename: Response to reviewers_ScR Features of Mob Apps for DKD Pt.pdf [file pone.0331604.s005.pdf]

Rebuttal Letter: Responses to Editor/Journal requirements and Reviewers

Dear Academic Editor and Reviewers,

Thank you for your valuable feedback on our manuscript (ID: PONE-D-25-44415). We have carefully considered all comments and revised the manuscript accordingly. Below is a point-by-point response to each comment which divided into;

A. Editor-journal requirements
B. Responses to reviewers

A. Editor-journal requirements

1. Please ensure that your manuscript meets PLOS ONE's style requirements, including those for file naming.

   Response: The manuscript has been checked at the website provided and complies with the PLOS ONE template, formatting, and file-naming requirements. Parts included are;
   i. Author, affiliations formatting
   ii. Removed funding related texts

2. Please provide an amended statement that declares *all* the funding or sources of support (whether external or internal to your organization) received during this study, as detailed online in our guide for authors at http://journals.plos.org/plosone/s/submit-now. Please also include the statement "There was no additional external funding received for this study." in your updated Funding Statement.
   Please include your amended Funding Statement within your cover letter. We will change the online submission form on your behalf.

   Response: We have included the revised funding statement in the cover letter.

3. Authors must share the "minimal data set" for their submission. PLOS defines the minimal data set to consist of the data required to replicate all study findings reported in the article, as well as related metadata and methods.
   For example, authors should submit the following data:
   - The values behind the means, standard deviations and other measures reported;
   - The values used to build graphs;
   - The points extracted from images for analysis.
   Authors do not need to submit their entire data set if only a portion of the data was used in the reported study.

   Response:
   Thank you for highlighting the journal's data availability requirements. As this study is a scoping review, no primary or individual-level data were collected. In accordance with PLOS ONE's definition of a minimal data set, we have provided the extracted study-level

data used to generate all tables and figures as Supporting Information (S1 Dataset). This dataset enables replication of all reported findings.

4. Please remove any funding-related text from the manuscript and let us know how you would like to update your Funding Statement. Currently, your Funding Statement reads as follows:
   "This work was supported by the Universiti Sains Malaysia, Research University Team (RUTeam) Grant Scheme with Project No: 1001/PPSP/8580083, Project Code: TE0034 (Reference No: 2022/0501]
   The funding provided covered the author's role as a research assistant and facilitated access to the Universiti Sains Malaysia library's electronic databases and resources"
   Please include your amended statements within your cover letter; we will change the online submission form on your behalf.

   Response: The revised Funding Statement is included in the cover letter and has been removed from the manuscript.

5. The absence of adequate discussion of the results with appropriate references has watered down the reproducibility of the methodologies, results and the overall chance for publication of the current submission.
   Additionally, the authors need to justify the significance of the study, relate it to the literature and identify the gap in the existing knowledge that this study aims to address. Again, what are the limitations of this study, and how can the authors recommend future research on the study? Moreover, some other major concerns have been raised by the reviewers affecting pivotal sections of the study. Kindly pay close attention to these and address them critically before resubmission.

   Response: as per responses to the reviewers below.

B. Responses to the reviewers

Reviewer #1

1.1. **Comment**: After reviewing your manuscript, I found that it follows the template and meets the PLOS ONE style requirements, including file naming.

Response: We thank the reviewer for confirming that the manuscript complies with the PLOS ONE template, formatting, and file-naming requirements.

1.2. **Comment**: The presentation of research data should be well-synthesized, with the aim of summarizing the number of pages and displaying it with attractive tables and graphs, following the PLOS ONE template.
Therefore, authors should further enhance their literacy by reading high-impact journals, as they reflect the quality of their research and publications.

Response:
Thank you for this thoughtful comment. We agree that effective synthesis and clear presentation of research data are essential for high-quality reporting. In the current manuscript, the included studies have been summarized using structured tables in accordance with the PLOS ONE guidelines, with the aim of presenting key characteristics and findings in a concise and transparent manner.

The manuscript has been revised to further improve data synthesis and presentation in line with the PLOS ONE template. Specifically, the Results section was streamlined, redundant text reduced, and key findings clearly summarized using tables and graphical representations, as appropriate.

After careful consideration, we did not add additional tables or figures, as further graphical presentation would be largely redundant and unlikely to enhance clarity. We believe that the existing tables sufficiently synthesize the evidence and align with the expectations for scoping reviews published in PLOS ONE.

Reviewer #2

2.1 **Comment**: Grammatical error and Language Inconsistency
Please ensure tense consistent (kindly review every section of this work with this point in mind), correct few grammatical errors, and vet included sentences for redundancies.

Response:
We have carefully reviewed the manuscript in full to ensure consistent tense use, corrected grammatical errors, and edited redundant or repetitive phrasing for clarity and scientific tone. These revisions were implemented across the Introduction and Methods sections, as well as other relevant parts of the manuscript. A concise summary of these language and editorial revisions is provided in Appendix A.

2.1.1 "However, because DKD is closely linked to both diabetes and CKD, mobile apps built for either condition often include overlapping features..."
Concern: Overly repetitive of the idea in subsequent sentences.

Response:
Introduction, paragraph 4 (line 81 of the original manuscript)
"Because DKD shares key self-management needs with both diabetes and CKD, mobile apps developed for either condition often include features relevant to DKD management, such as blood glucose and blood pressure monitoring, medication reminders, educational content, and provide communication tools".

2.1.2 "Looking across all three conditions helps provide a complete picture..."

Concern: "Looking across..." Please use a scientific language.

Response:
Introduction, paragraph 5 (Line 93 of the original manuscript)
“Examining mobile applications across these three interrelated conditions provides a more comprehensive understanding of how mobile technology supports key self-management behaviours, including monitoring, education, motivation, and treatment adherence.”

2.1.3 “Studies was included if they describe...”
Concern: Review the your choice of verbal in relation to the plural subject, "Studies".
Response:
Materials and Methods – Inclusion criteria, paragraph 1 (lines 126 of the original manuscript)
“Studies were included if they described or evaluated mobile applications (apps) designed to support self-management among individuals with diabetic kidney disease (DKD), diabetes, or chronic kidney disease”.

2.1.4 “Two reviewers have independently screened...”
Concern: You inclusion of "have" was not necessary.

Response:
Methods – Study selection- paragraph 1(line 164 of the manuscript)
Two reviewers independently screened titles and abstracts against the inclusion criteria.

2.1.5 “Table 3 shows four recurring core functional domains of self-management features were identified…”
Concern: Awkward phrasing.
Response:
Results – Core functional domains (Line 254 of the manuscript)
Table 3 summarizes four recurring core functional domains of self-management features identified across the included studies.

In addition to the specific issues highlighted by the reviewer, the manuscript was systematically reviewed for other grammatical errors, tense inconsistencies, redundancy, and awkward phrasing. A summary of these additional language revisions is provided in Appendix A.

2.2 **Comment**: Lengthy Discussion section.
I consider the discussion paragraphs unnecessarily lengthy and wordy. Kindly condense it.

Response:
In response, we have revised the Discussion section to make it clearer and easier to read. Long paragraphs were shortened, repeated explanations were removed, and unnecessary

wording was reduced. The revised Discussion now focuses more on interpreting the findings rather than repeating the results, while keeping the main messages of the study.

2.3 **Comment**: The claim in this statement, "Our study is among the first to comprehensively explore mobile health apps spanning DKD, T2DM, and CKD." is considered an overstatement because of your use of the word "first". Please, substitute with other words, e.g. "few".

Response:
We have revised the wording to remove the term "first" and ensured that the Discussion reflects a more conservative and accurate description of the study's contribution. No priority claims are made in the revised manuscript.

Reviewer #3

3.1.1 **Comment**: Methodology- Issue 1: Absence of pilot testing which has affected Reproducibility.
Refer to this statement, "Data charting was conducted using a standardised extraction form developed by the review team, which included predefined fields based on the review objectives and PCC framework."
Recommendation: Please include a well coordinated pilot testing or validation to enhance reproducibility.

Response:
According to the JBI Manual for Evidence Synthesis, data charting in scoping reviews is an iterative process, and while charting forms may be piloted on a small number of studies, they may also be refined during the review process as familiarity with the literature increases. In line with this guidance, the data extraction form in our review was developed based on the PCC framework and iteratively tested and refined during the initial stages of data charting to ensure consistency and reproducibility. This clarification has now been explicitly added to the Methods section.

3.2 **Comment**: Methodology- Issue 2: Data synthesis.
Recommendation: Please include a description of how features were categorized into the four functional domains.

Response:
We have now added a clear description of the data synthesis process in the Methods section, explaining how mobile app features were categorised into four functional domains based on their primary function, how overlapping features were handled, and how consensus was achieved among the review team.

3.3 **Comment**: Discussion- Issue 1: Structural concern.
I consider this section's paragraphs to be too long.
Recommendation: Please split this by theme; e.g. Self-management features, Integration with care teams, Regional variations, Limitations and implications. This will potentially increase depth of discussion but you do need to avoid wordiness.

Response:
We agree with the reviewer and have comprehensively restructured the Discussion section. Clear thematic subheadings have been introduced, including:

i. Principal findings
ii. Self-management feature domains
iii. Supportive devices and integration with care teams
iv. Regional and contextual variation
v. Outcome reporting versus effectiveness
vi. Data security and ethical considerations
vii. Limitations
viii. Implications for app development and clinical practice

This reorganization improves logical flow, enhances thematic depth, and ensures that each paragraph addresses a single coherent concept.

3.4 **Comment**: Discussion- Issue 2: Shallow limitation and Implications discussion.
Recommendation: Kindly include a subsection that will summarize the implications of your study for app developers and clinical practice. Similarly, expand on limitations of database coverage.

Response:
We appreciate this suggestion and have expanded the Limitations section to explicitly address database and language restrictions, potential exclusion of grey literature (e.g., app store and industry reports), lack of quality appraisal, and the feature-focused scope of the review. In addition, a new subsection on implications has been added, outlining practical considerations for app developers, clinical integration, and future research directions. These revisions demonstrate broader critical reflection and practical relevance.

3.5 **Comment**: Discussion- Issue 3: Data security and ethical implications.
Recommendation: Please expand on how data security and ethical concerns can be safely protected.

Response:
Thank you for highlighting this important issue. A dedicated subsection on data security and ethical considerations has been added to the Discussion. This section now addresses informed consent, data minimization, secure data storage and transmission, access

controls, transparency in data use, and equity considerations for users with varying levels of digital literacy. Although detailed security reporting was limited in the included studies, this addition strengthens the ethical framing of the review.

3.6 **Comment**: Discussion- Issue 4: Minor tautology.
"Interactive, behaviorally informed, and patient-centred app design"
Recommendation: Kindly omit one of "behaviorally informed" and "patient-centred" as they have semantic overlap.

Response:
We have revised this phrase to remove redundancy. The wording has been simplified to "interactive and patient-centred app design," thereby improving clarity and precision.

3.7 **Comment**: Discussion- Issue 5: Potential Inconsistency.
Earlier, you stated under the subheading, Integration with Care team and Outcome Measure: "The included studies reported a range of outcome measures encompassing adherence metrics (such as medication compliance and goal tracking), biometric improvements (including blood pressure, HbA1c, and weight), and patient-reported outcomes related to usability and satisfaction [11,21]". However, this statement in the Discussion section, "Although outcomes such as adherence, glycemic control, or usability were reported in several studies, this review did not evaluate their effectiveness" can be seen as a contradiction. This is because the indices alluded to are key effectiveness indicators.

Response:
We have revised the wording in the discussion section to make it clearer. While several studies reported outcomes such as adherence, glycaemic control, and usability, this scoping review did not assess or compare the effectiveness of the interventions because the study designs, outcome measures, and follow-up periods were very different. This clarification follows scoping review methodology and addresses the concern raised.

3.8 **Comment**: Grammatical Errors.
I encourage you to go through the whole manuscript and carefully look out for typographical errors, missed areas of punctuation, wordiness, awkward phrasing and overstatement with the aim of correcting them.

Response:
The manuscript was systematically reviewed for other grammatical errors, tense inconsistencies, redundancy, and awkward phrasing. A summary of these additional language revisions is provided in Appendix A.

Reviewer #4

4.1 **Comment**: In table 4, kindly stay consistent with how your sentences are written; that is, are you using lowercases all through or title cases?

Response:

We have revised Table 4 and all other tables to ensure consistent sentence case formatting throughout the table.

4.2 **Comment**: Ensure consistency. spell check: there was a point where the word "through" is written as "thru".

Response:

The manuscript was systematically reviewed for other grammatical errors, tense inconsistencies, redundancy, and awkward phrasing. A summary of these additional language revisions is provided in Appendix A.

4.3 **Comment**: Lastly, what are the other methods/frameworks for conducting scoping reviews, and what influenced your decision for choosing the framework that was used in this review?

Response:

Several methodological frameworks are available for conducting scoping reviews, including the original framework proposed by Arksey and O'Malley and subsequent methodological enhancements by Levac et al.

In this review, we selected the Joanna Briggs Institute (JBI) methodology because it provides comprehensive and updated guidance specifically tailored for scoping reviews, including the use of the Population–Concept–Context (PCC) framework, structured data charting, and transparent synthesis of evidence. The JBI approach is also closely aligned with the PRISMA-ScR reporting guidelines and is widely adopted in contemporary health and digital health research. This rationale has now been clarified in the manuscript.

Reviewer #5

5.1 **Comment**: including data on downloads or active users would show which apps are truly engaging and have a proven track record.

Response:

Thank you for this valuable suggestion. We agree that metrics such as download counts, active users, and engagement duration would provide important insight into real-world app uptake. However, such data were not consistently reported in the included peer-reviewed studies and are often available only through app store analytics or proprietary sources. We have now explicitly acknowledged this gap as a limitation and highlighted the need for future research to incorporate real-world usage and engagement metrics.

5.2 **Comment**: clarifying the cost models of the reviewed apps (e.g., free, freemium, one-time purchase, subscription-based) is essential for assessing equity and practical utility. This information could be added as a new section in the results, or discussed as a key point for

future research in the conclusion. Acknowledging this as a limitation also shows the authors have thought carefully about the bigger picture.

Response:

We agree with the reviewer that cost models play an important role in accessibility, equity, and practical use of mobile health apps.. However, pricing information was rarely reported in the included studies and could not be systematically extracted. To address this, we have acknowledged the lack of cost transparency as a limitation and discussed its implications for equity and future research in the Discussion and Conclusion sections.

5.3 **Comment**: A minor editorial point was noted regarding citation style. It is recommended to use an en dash (–) for ranges of three or more consecutive numbers (Line # 172, #207, #250, #255).

Response:

Suggestion accepted and changed made.
Originally Line # 172 --------------- #192
Line #207 -----------------------------#229
Line #250, -------------------------------#276
Line#255)----------------------------#281

We appreciate your time and effort in reviewing our work. We believe the revisions have strengthened the manuscript significantly. Please let us know if further clarifications are needed. A marked-up version of the manuscript with tracked changes has been provided, alongside a clean revised manuscript.

Sincerely,

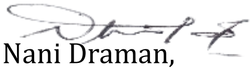

Nani Draman,
Corresponding Author
Universiti Sains Malaysia,

Appendix A. Summary of Additional Language Revisions

Section	Location (Paragraph–Sentence)	Issue Identified	Amendment Made
Introduction	Para 3, Sentence 2	Typographical and punctuation error	Added missing space after full stop and inserted comma after "chronic kidney disease".
Introduction	Final paragraph, Sentence 3	Informal / wordy phrasing	Replaced informal expressions ("hopes to highlight", "doing well") with academic wording ("aims to highlight", "well established").
Introduction	Final paragraph, Sentence 2	Typographical error	Removed double punctuation ("..")
Materials and Methods	Section heading	Heading inconsistency	Revised heading from "Material and methods" to "Materials and methods".
Materials and Methods	Eligibility criteria paragraph, Sentence 1	Subject–verb agreement	Corrected plural verb usage ("Studies was" → "Studies were").
Results	Throughout Results text	Verb tense inconsistency	Standardised all descriptions of findings to past tense.
Discussion	Interpretation paragraphs	Overstatement	Softened strong claims (e.g., "clearly demonstrates" → "suggests").
Limitations	Section heading	Heading inconsistency	Revised heading from "Limitation" to "Limitations".
